# Cryogenic Single-Molecule Fluorescence Detection of the Mid-Infrared Response of an Intrinsic Pigment in a Light-Harvesting Complex

**DOI:** 10.1021/acs.jpcb.3c00284

**Published:** 2023-05-24

**Authors:** Kohei Otomo, Takehisa Dewa, Michio Matsushita, Satoru Fujiyoshi

**Affiliations:** † Department of Physics, Tokyo Institute of Technology, Meguro, Tokyo 152-8550, Japan; ‡ Department of Biochemistry and System Biomedicine, Graduate School of Medicine, Juntendo University, Bunkyo, Tokyo 113-8421, Japan; § Department of Life Science and Applied Chemistry, Graduate School of Engineering, Nagoya Institute of Technology, Nagoya 466-855, Japan

## Abstract

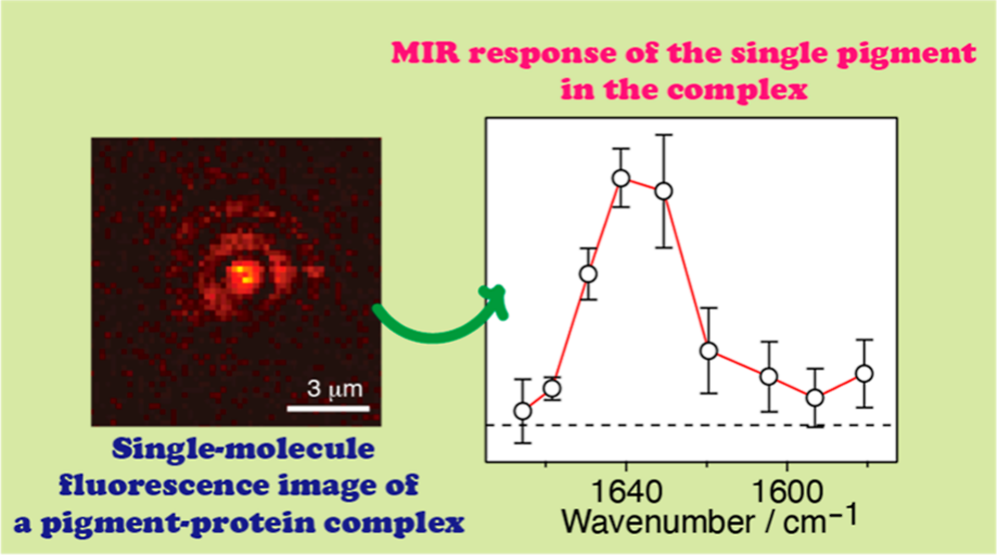

We observed the mid-infrared (MIR) response of a single pigment of bacteriochlorophyll *a* at the B800 binding site of a light-harvesting 2 complex. At a temperature of 1.5 K, a single complex in a spatially isolated spot in a near-infrared (NIR) fluorescence image was selected and was simultaneously irradiated with MIR and NIR light. We found that the temporal behavior of the NIR fluorescence excitation spectrum of individual pigments in a single complex was modulated by the MIR irradiation at 1650 cm^–1^. The MIR modulation of a single pigment was linearly proportional to the MIR intensity. The MIR linear response was detected in the range from 1580 to 1670 cm^–1^.

## 1 Introduction

Mid-infrared (MIR) spectroscopy is a useful analytical tool in protein science, with applications ranging from measuring protein content in aqueous solutions to studying protein structures in biological systems.^1−17^ In particular, the MIR absorption of the C=O stretching vibration in the range from 1600 to 1700 cm^–1^ (approximately 6 μm in wavelength) is sensitive to the secondary structures of protein-backbones and the hydrogen bonding network inside the proteins.^1,13−16^ For example, neighboring C=O vibrations in the backbone couple with each other to form coupled vibrations (amide-I band);^1−7^ thus, the formation of secondary structures, such as α-helices and β-sheets, shifts the center wavenumber of the amide-I band. However, structural analysis is often limited by the broad spectral feature of the C=O absorption band. The broadening of the C=O band is caused by overlap between different C=O vibrations. Since proteins contain the same number of backbone C=O groups as amino acids, the amide-I region of the vibrational spectrum of proteins consists of hundreds of C=O bands overlapping each other. In addition to the intramolecular heterogeneity of the C=O vibrations, the intermolecular heterogeneity of protein conformations contributes to the broadening of vibrational bands. With the current sensitivity of a conventional Fourier transform infrared (FTIR) spectrometer, a minimum of approximately 10^6^ molecules is necessary for a detectable signal. Conformational heterogeneity, which is considered important for biological functions, cannot be detected due to the ensemble averaging over >10^6^ different molecules.

To develop an optical method for studying the vibrations of individual proteins, we investigated the effect of the MIR influence on the photoinduced cycling of a dye between its fluorescent and nonfluorescent states.^18^ A dye (Alexa Fluor 660, AF660) was bound to a protein (bovine serum albumin, BSA) via a linker a few nanometers in length; thus, AF660 remained outside of BSA. Visible irradiation at 632 nm often bleached the AF660 molecule even at a few Kelvin,^19,20^ and the MIR irradiation at 1650 cm^–1^ caused the photobleached AF660 molecule to recover its fluorescence.^18^ The MIR response of the dye was obtained by measuring the steady-state population ratio between the fluorescent and nonfluorescent states as a function of the MIR wavenumber. The MIR response spectrum of a single BSA molecule was in agreement with the FTIR spectrum of the amide-I absorption of the ensemble. This indicated that regardless of which vibration absorbed the MIR photons, the vibrational energy affected the AF660 molecule with a similar efficiency. The opposite situation, in which the C=O groups are different in terms of the strength of the MIR effect, occurs if the dye is located inside the protein. The initial processes of a vibrational energy flow taking place inside a protein are known to be strongly dependent on the distance from a photon-absorbing group.^21−23^ Since some proteins have native fluorescent chromophores,^24^ we expect to observe a dependence of the fluorescence of these intrinsic chromophores on the MIR excitation of vibrational modes in their surrounding environments.

In the present paper, we applied cryogenic single-molecule fluorescence microscopy to an intrinsic pigment at the binding site of a pigment–protein complex to obtain the MIR response of this pigment. The signals of individual bacteriochlorophyll *a* (BChl-*a*) pigments in light-harvesting 2 (LH2) complex are known to appear as sharp peaks in NIR fluorescence excitation spectra at a few Kelvin.^25,26^ The peak positions of the individual pigments discontinuously change among a few distinct frequencies, which is referred to as spectral jump.^27,28^ The temporal behavior of a single BChl-*a* pigment at 1.5 K was found to be modified by MIR irradiation at 1650 cm^–1^. The MIR modulation of the temporal behavior of a single pigment was linearly proportional to the MIR intensity. The MIR response spectrum of the single BChl-*a* pigment in the LH2 complex was different from the FTIR spectrum of its ensemble; the bandwidth was roughly halved, and the peak was redshifted from 1650 to 1637 cm^–1^.

## 2 Methods

### 2.1 Sample for Cryogenic Fluorescence Microscopy

**Figure 1:**
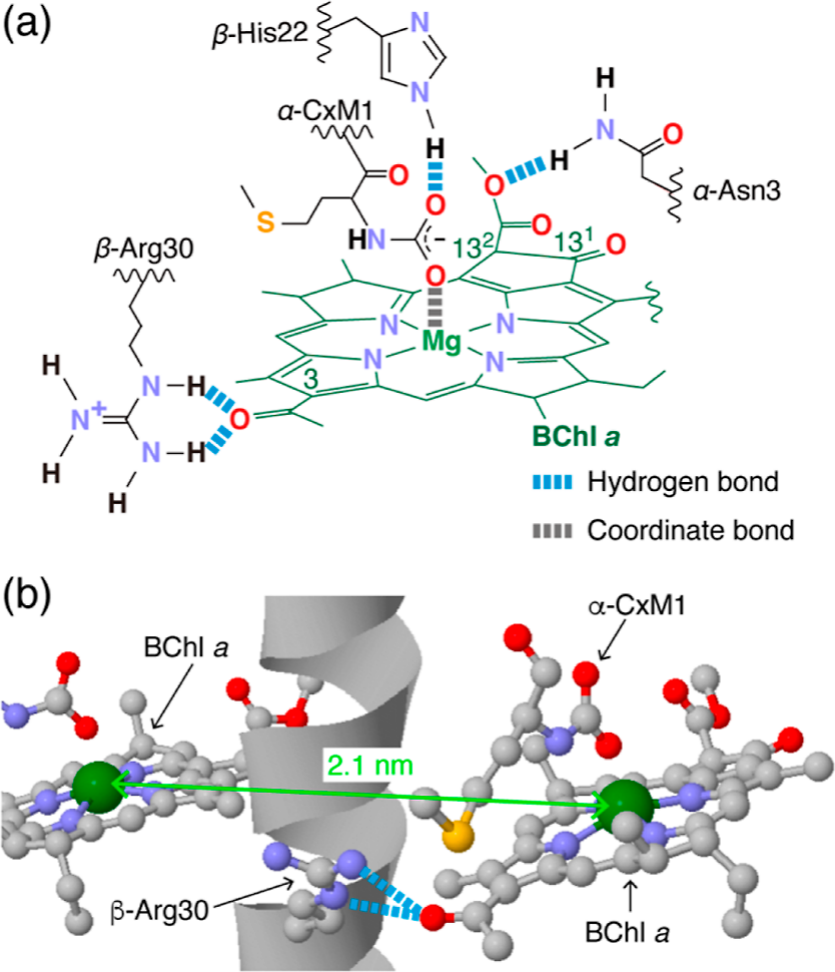
Structure of the B800 binding site of the LH2 complex from *R. sphaeroides*. The binding site is represented by its chemical formula (a) and a ball and stick model (b). Light blue and gray dashed lines indicate hydrogen bonds and coordinate bonds, respectively. The colors denote the following elements as follows: red = oxygen, gray = carbon, blue = nitrogen, green = magnesium, and yellow = sulfur. The gray ribbon represents the α-helix of the β-polypeptide of the LH2 complex.

The LH2 complex was isolated from the purple photosynthetic bacterium *Rhodobacter* (Rb.) *sphaeroides* 2.4.1 with a previously reported method.^29^Figure S1 shows the NIR absorption spectrum of the LH2 complex in a D_2_O buffer solution (5 × 10 ^–8^ M) at 297 K. The ratio between the absorbance values at 280 nm (protein) and 800 nm (the B800 pigments) was 0.38, and the ratio between the absorbance values at 850 and 800 nm (the B800 pigments) was 1.4. The ratio values were consistent with the expected stoichiometry in the LH2 complex.^30,31^Figure 1 shows the structure of the B800 binding site of the LH2 complex.^32^ The LH2 complex has nine B800 binding sites arranged in a circle, and one BChl-*a* pigment bonded to each binding site via three hydrogen bonds and a coordinate bond (Figure 1a). Since the individual BChl-*a* pigments were separated by 2.1 nm, their electronic states were localized to each pigment.

### 2.2 NIR Imaging of Individual LH2 Complexes at 1.5 K and the Spectroscopy of Their B800 Band

**Figure 2:**
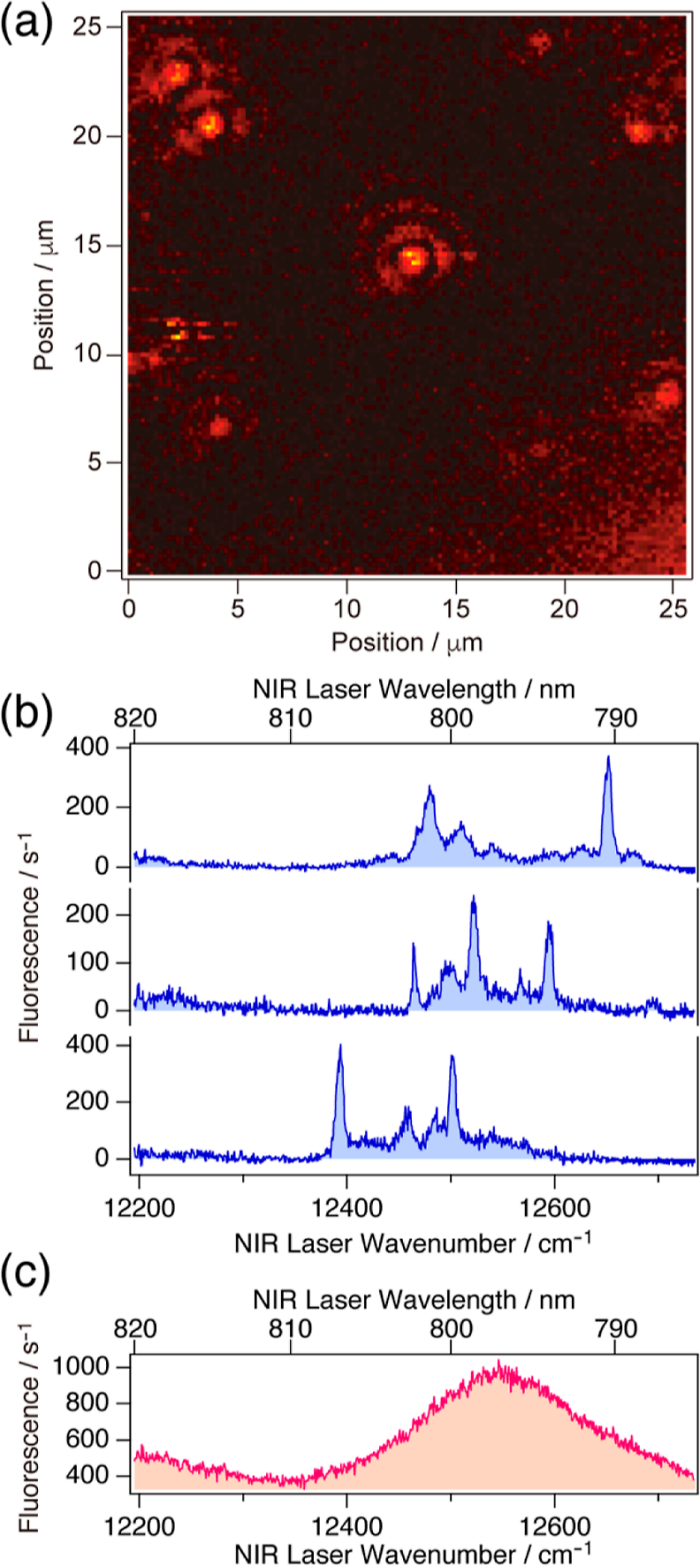
NIR fluorescence excitation spectra of individual LH2 complexes. (a) NIR fluorescence image of individual LH2 complexes at 1.5 K by raster laser scanning. The laser wavelength was 835 nm; hence, every LH2 complex was excited in the B850 band. Ten LH2 complexes are present in the image. (b) Three examples of the fluorescence excitation spectra of a single LH2 complex at 1.5 K. (c) Fluorescence excitation spectrum of a single LH2 complex at 80 K. Note that the spectra of the other complexes at 80 K were similarly broad.

Figure 2a shows a laser-scanning image of the near-infrared (NIR) fluorescence of individual LH2 complexes at 1.5 K. The excitation laser wavelength was 835 nm (11,980 cm^–1^), which resonated with the electronic absorption of the B850 band of the LH2 complex. The excitation of the B850 band delocalized over 18 BChl-*a* pigments that were arranged in a circle. For a single LH2 complex at a few Kelvin, the fluorescence excitation spectrum of the B850 band was as broad as the ensemble spectrum;^25,33^ therefore, all LH2 complexes in an observation area were able to be exhaustively imaged even by a single-wavelength laser light with a bandwidth of 1 cm^–1^. A sample for microscopy was prepared by spin-coating a D_2_O buffer solution containing a detergent-solubilized LH2 complex and 1% wt/wt poly(vinyl alcohol) on a CaF_2_ substrate. The sample prepared from the 10^–11^ M solution of the LH2 complex resulted in a spatial density that allows each LH2 complex to appear as a spatially isolated diffraction pattern with a diameter of the first dark ring of 1.7 μm.

The fluorescence excitation spectrum of a single LH2 complex was acquired in two steps: (1) selection of a spatially isolated spot in the fluorescence image obtained by exciting the B850 band at 835 nm (see Figure 2a) and (2) measurement of the fluorescence intensity in the wavelength range from 785 to 820 nm (from 12,750 to 12,200 cm^–1^) while scanning the NIR laser frequency through the B800 band. Figure 2b shows the fluorescence excitation spectra of several individual LH2 complexes at 1.5 K. Because the nine BChl-*a* pigments in the equivalent B800 binding sites were circularly arranged with the nearest-neighbor Mg-to-Mg distance of approximately 2.1 nm (Figure 1b), in the spectrum of a single LH2 complex at a few Kelvin, the B800 band basically consists of nine independent peaks, each of which corresponds to a local electronic excitation of one of the nine BChl-*a* pigments.^34,35^ In the present experiment, because a linearly polarized NIR laser was used, approximately half of the 9 BChl-*a* pigments were observed as spectral peaks with a full width at half maximum (fwhm) of 4–12 cm^–1^. Figure 2c shows the fluorescence excitation spectrum of a single LH2 complex at 80 K. The B800 band of a single LH2 complex at 80 K appeared as a broad featureless band. When the temperature is increased from 1.5 K to approximately 20 K, the sharp peaks of single LH2 complexes were broadened,^36^ indicating that the thermally activated structural changes of the protein became more frequent.

### 2.3 Cryogenic Fluorescence Microscope

The setup of the cryogenic microscope employed here was based on a previously reported setup.^18^ The key optic was a cryogenic objective mirror made of CaF_2_. The objective consisted of two spherical internal mirrors^18,20,37−39^ made of a single piece of CaF_2_. When light rays propagating in parallel in superfluid helium entered the CaF_2_ body of the objective mirror, the rays were internally reflected twice at the interface, left the CaF_2_ body, and were focused to a diffraction-limited spot. The CaF_2_ body material transmitted light from the ultraviolet through the MIR region, and the objective worked achromatically. To halve the spherical aberration,^38^ the size of the CaF_2_ objective mirror (focal length of 2 mm and the numerical aperture of 0.6) was made half that of the previous version of the objective (focal length of 4 mm and numerical aperture of 0.6).^18^ As a result, the fluorescence spot nearly reached the diffraction limit (see Figure 2a). An NIR fluorescence image was obtained by raster-scanning the focus of the excitation light (while the sample was fixed in superfluid helium). The fluorescence of the LH2 complex was filtered by bandpass filters (the window from 850 to 920 nm) and detected by an avalanche photodiode (SPCM-AQR-16FC, PerkinElmer). The total detection efficiency of the NIR fluorescence was approximately 1%. The NIR light source for the electronic excitation of BChl-*a* at the B800 band and the B850 band was a continuous-wave-tunable Ti:sapphire laser (899, Coherent) with a bandwidth of 1 cm^–1^ and a focal point intensity of 10–75 W cm^–2^. At the absorption cross-section of the B800 band of 3 × 10^–15^ cm^2^, a typical intensity of 20 W cm^–2^ corresponded to an NIR-photon absorption rate of 2 × 10^5^ s^–1^ for the LH2 complex. For fluorescence imaging and spectroscopy, the NIR laser light was spectrally cleaned with a shortpass filter (FF01-842/SP, Semrock) for the B800 band observation and a bandpass filter (FF01-794/160, Semrock) for the B850 band observation. The fluorescence signal was separated from the NIR light with longpass filters, FF01-835/LP (Semrock) for B800-band observation and LIX870 (Asashi Spectra) for B850-band observation.

The MIR light source for vibrational excitation was circularly polarized with a 500 ns pulse from a quantum cascade laser that was tunable from 1580 to 1670 cm^–1^ (Daylight Solutions). The maximum pulse-repetition rate of 100 kHz yielded a time-averaged intensity of 890 W cm^–2^. The duty cycle was 0.05. From the intensity and the duty cycle, the maximum photon flux density at the focus was estimated to be 5 × 10^23^ s^–1^ cm^–2^. At 1.5 K, the absorption cross-section of the LH2 complex at 1650 cm^–1^ was determined by FTIR measurements to be 9 × 10^–15^ cm^2^. From the photon flux density and the cross-section, the MIR photon absorption rate of the LH2 complex was estimated to be 5 × 10^9^ s^–1^, corresponding to a time interval of the MIR photon absorption of 200 ps. The vibrational energy inside the protein relaxed on a timescale of 1–20 ps.^40−42^ The time interval of the MIR photon absorption was an order of magnitude longer than the relaxation time of the vibrational energy; consequently, the energy from a MIR photon dissipated away from the LH2 complex before the next photon was absorbed. The MIR irradiation of the LH2 complex did not give rise to nonlinear multiphoton processes.

### 2.4 FTIR Spectroscopy of the LH2 Complex at 1.5 K

FTIR spectroscopy of an ensemble of the LH2 complex was carried out with a commercial FTIR spectrometer (Nicolet 6700, Thermo Fisher Scientific, USA), a custom-made cryostat, and additional optics. The measurement was carried out in the transmission configuration. The sample plate was prepared by dip-coating a CaF_2_ substrate with a D_2_O buffer solution containing a detergent-solubilized LH2 complex and 1% wt/wt poly(vinyl alcohol). The plate was immersed in superfluid helium in the custom-built cryostat^43^ that was used for the microscopy measurements.

## 3 Results

### 3.1 MIR Effect on the NIR Fluorescence Excitation Spectra of Individual BChl-*a* Pigments

**Figure 3:**
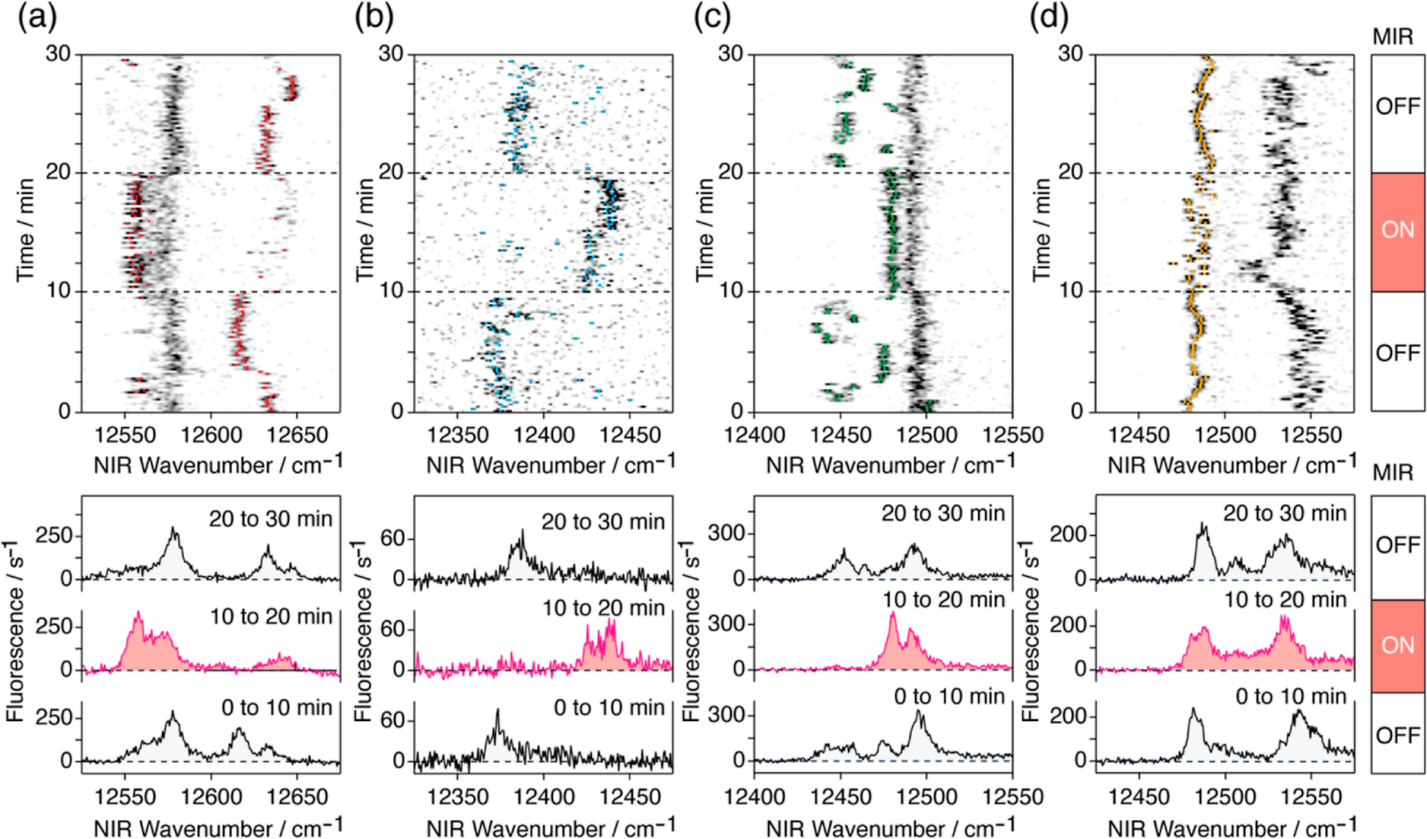
MIR influence on the fluorescence excitation spectra of individual BChl-*a* pigments at the B800 site at 1.5 K. The upper panel presents a grayscale two-dimensional plot of the fluorescence intensity as a function of the NIR laser wavenumber and time along the horizontal and the vertical axes, respectively. The wavenumber of the NIR laser was scanned at a rate of 65 cm^–1^ s^–1^. During the period from 10 to 20 min, the sample was irradiated by a pulsed MIR laser at a wavenumber of 1650 cm^–1^ and an intensity of 890 W cm^–2^. The lower panel presents the spectra averaged over periods from 0 to 10, 10 to 20, and 20 to 30 min.

Figure 3 shows some examples of the MIR irradiation effect on the fluorescence excitation spectra of individual BChl-*a* pigments in the B800 binding site of the LH2 complex at 1.5 K. The upper panels show the time course of the fluorescence excitation spectra of single BChl-*a* pigments in a 2D representation with the time and NIR wavenumber along the vertical and horizontal axes, respectively. The MIR light at 1650 cm^–1^ was switched on at 10 min and switched off at 20 min. The peak positions of single BChl-*a* pigments were determined for each NIR frequency scan by a fitting analysis and are represented by color lines. The lower panels of the figure show time-averaged spectra over three periods: before, during, and after MIR irradiation. The spectral response to the MIR light is summarized as follows.

Signals from two BChl-*a* pigments are shown in Figure 3a. In the first 10 min, when the MIR light was off, the signal of the molecule marked by red stayed above the NIR laser wavenumber of 12,610 cm^–1^ for the majority of the time. At the moment of MIR irradiation, the signal redshifted and stayed at approximately 12,560 cm^–1^ for the majority of the time until the MIR light was switched off. After the MIR light was switched off, the signal stayed above 12,610 cm^–1^. The signal of the other BChl-*a* pigment stayed at approximately 12,580 cm^–1^ to give rise to an average spectrum with a fwhm of approximately 15 cm^–1^ when the MIR light was turned off (in the first period from 0 to 10 min and in the third period from 20 to 30 min). When the MIR light was switched on (in the second period from 10 to 20 min), the instantaneous peak slightly shifted toward a lower wavenumber, and the fwhm of the average spectrum approximately doubled. In Figure 3b, the signal from a single BChl-*a* pigment is marked by light blue. The moment the MIR light was turned on at 10 min, the spectral peak blueshifted from 12,370 to 12,440 cm^–1^. When the MIR light was turned off at 20 min, the spectral peak returned to the original wavenumber of 12,370 cm^–1^. Figure 3c shows the spectra for two BChl-*a* pigments. When the MIR light was off, the spectral peaks of the molecules were approximately 12,450 and 12,490 cm^–1^. The former, marked by green, responded to the MIR light, whereas the latter did not and stayed at approximately 12,490 cm^–1^ the entire time. Without MIR irradiation, the peak marked by green made spectral jumps among several positions between 12,430 and 12,480 cm^–1^. When the MIR light was on, it stayed at 12,480 cm^–1^. Signals from two BChl-*a* pigments are shown in Figure 3d; one, starting at 12,550 cm^–1^, made spectral jumps, but the temporal behavior was not correlated with MIR irradiation. Another BChl-*a* pigment marked by yellow stayed at approximately 12,480 cm^–1^ irrespective of MIR irradiation. However, the rate at which the instantaneous peak jumped approximately 10 cm^–1^ increased when the MIR light was on.

### 3.2 MIR Response Spectrum of a Single BChl-*a* Pigment at the B800 Binding Site of the LH2 Complex

**Figure 4:**
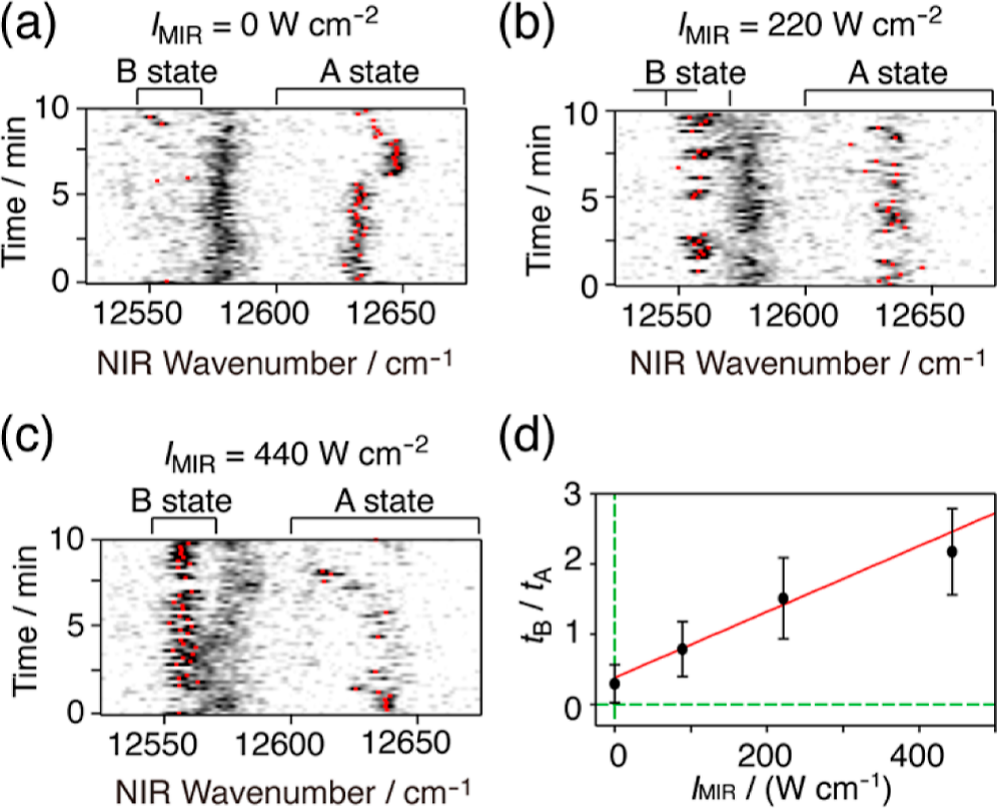
MIR-intensity (*I*_MIR_) dependence of the residence time in state A (*t*_A_) and state B (*t*_B_) of the single BChl-*a* pigment at 1.5 K denoted by the red color in Figure 3a. (a–c) Time course of the fluorescence excitation spectrum determined at *I*_MIR_ = 0, 220 and 440 W cm^–2^, respectively. (d) Residence–time ratio *t*_B_/*t*_A_ plotted against *I*_MIR_. The red straight line is the result of the linear fitting, *t*_B_/*t*_A_ = 0.21 + 0.0047 *I*_MIR_/(W cm^–2^).

**Figure 5:**
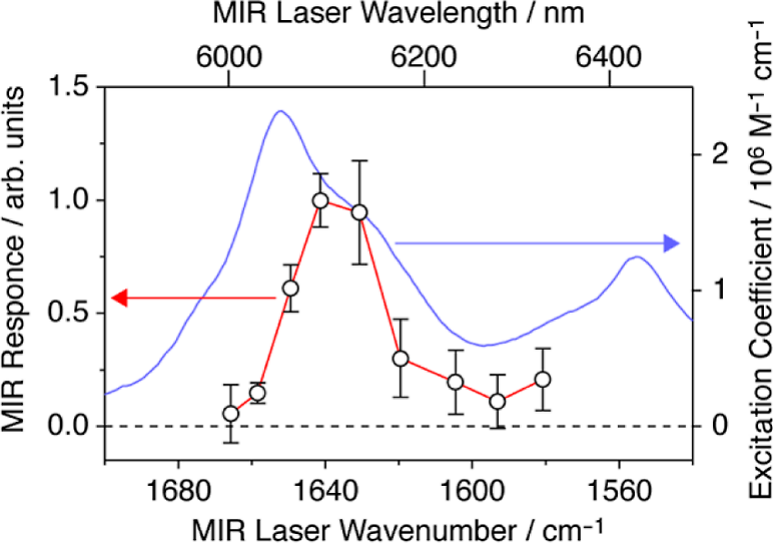
MIR response spectrum of the single BChl-*a* pigment at 1.5 K denoted by the red color in Figure 3a. The MIR response was determined as (*t*_B_/*t*_A_ – 0.21) ν_MIR_/*I*_MIR_. The MIR intensity at the focal point was 350 W cm^–2^ and varied within ±25%. The blue curve indicates the FTIR spectrum of an ensemble of LH2 complexes determined at 1.5 K.

For the BChl-*a* pigment marked by red in Figure 3a, the MIR effect at 1.5 K was further examined in relation to the MIR intensity (*I*_MIR_). Figure 4a–c shows the time course of the fluorescence excitation spectrum obtained at three different MIR intensities. In all of the three time courses, the signal of the molecule marked by red appeared in a range either above 12,620 cm^–1^ (state A) or below 12,565 cm^–1^ (state B), independent of whether the MIR light was on or off. The signal went back and forth between states A and B by making spectral jumps of a size exceeding the frequency gap between the two states. When increasing the MIR intensity, the signal tended to spend a longer time in state B and a shorter time in A. To simplify the analysis, we concentrated on the spectral jumps between states A and B, ignoring the other temporal behaviors. The jump was characterized by the residence time in states A and B, and the MIR effect on the jump was quantified in terms of the ratio of the residence time in state B (*t*_B_) to that in state A (*t*_A_), *t*_B_/*t*_A_. Figure 4d shows *t*_B_/*t*_A_ measured at four different MIR intensities, *I*_MIR_, of 0, 90, 220, and 440 W cm^–2^. The plot of *t*_B_/*t*_A_ against *I*_MIR_ indicated a linear relation between *t*_B_/*t*_A_ and *I*_MIR_. The red line in the figure shows the result of the linear fitting, *t*_B_/*t*_A_ = 0.21 + 0.0047 *I*_MIR_/(W cm^–2^). Instead of directly observing the MIR-photon absorption of a single molecule, we observed the linear response of a single molecule to MIR light, i.e., the MIR-induced difference in the residence-time ratio between two states. As indicated in the Supporting Information, we analyzed the MIR response by introducing a simple kinetics model for the local structural change between states A and B. Briefly, the slope of *t*_B_/*t*_A_ (*t*_B_/*t*_A_ – 0.21) divided by the photon flux of MIR light (*I*_MIR_/*h*ν_MIR_) is mainly proportional to the absorption cross-section of state A. The MIR response, (*t*_B_/*t*_A_ – 0.21) *h*ν_MIR_/*I*_MIR_, was obtained from a single BChl-*a* pigment at nine different MIR frequencies (Figure 5). The MIR response spectrum shows a peak at 1637 cm^–1^ with a fwhm bandwidth of approximately 30 cm^–1^. Compared to that in the FTIR spectrum of the ensemble (blue curve), the bandwidth of the MIR response of the single BChl-*a* pigment was roughly halved, and its peak wavenumber was redshifted by approximately 15 cm^–1^.

## 4 Discussion

In the present experiment, a BChl-*a* pigment was used as a fluorescence probe for the C==O vibration of individual LH2 complexes. A large contribution to the fluorescence response of the C=O groups of the BChl-*a* pigment was expected. The BChl-*a* pigment had three C=O groups as its side groups, namely, C3 acetyl, C13^1^ keto, and C13^2^ acetyl carbonyl groups (for the positions, see Figure 1a). The wavenumbers of these three carbonyl groups are 1632 cm^–1^ (C3 acetyl), 1699 cm^–1^ (C13^1^ keto), and 1674 cm^–1^ (C13^2^ acetyl).^44^ The peak wavenumber at 1637 cm^–1^ of the MIR response spectrum shown in Figure 5 was most likely due to the C3 acetyl C=O stretching vibration. In addition to the dominant contribution from the C3 acetyl C=O group, the asymmetric shape and the broadening of the spectrum suggested minor contributions from other vibrations, including the skeletal C=C stretching of BChl-*a* (1610–1615 cm^–1^)^45^ and a guanidino group, (NH_2_)_2_C^+^NH–, of β-Arg30, which appears as a doublet in water (1633–1636 and 1672–1673 cm^–1^).^46^

The hydrogen bond of the C3 acetyl group is known to shift the NIR electronic absorption of the B800 band.^44^ The C3 acetyl group of BChl-*a* forms a hydrogen bond with the β-Arg30 residue. Following the site-directed mutation of the β-Arg30 residue, the B800 signal blueshifted from 800 nm for the wild type to 793–787 nm. The blueshift corresponds to the breakage of the hydrogen bond. It can be assumed that the BChl-*a* pigment still stays inside the binding pocket because the absorption of the free monomeric BChl-*a* in ether is further blueshifted to 771 nm. In the present experiment, the magnitude of the spectral jump (>70 cm^–1^) in the electronic absorption is comparable with half of the shift due to the complete breakage of the hydrogen bond (160 cm^–1^). It seems that the spectral jump between states A and B (Figure 4) corresponds to the reorganization of the three-dimensional structure of the hydrogen bond and its surroundings.

Finally, we discuss the qualitative behavior of the MIR response detection scheme. We have studied the MIR response of 224 LH2 complexes including the 4 complexes shown in Figure 3. For 13 out of the 224 complexes, some of the BChl-*a* pigments reversibly responded to MIR irradiation. Among the 13 complexes, 5 showed a large spectral shift of more than 50 cm^–1^ (see the shifts shown in Figure 3a–c), and the remaining eight complexes showed a small shift comparable to the spontaneous jump when the MIR light was off (see the shifts shown in Figure 3d). The manner in which the spectra change varies from pigment to pigment, indicating that among the many possibilities of structural change, which change occurs at what rate is determined by the structural details of the pigment-binding site. In addition, the spectral overlap with another molecule made it impossible to follow the identical molecule in time. This explains why only a small fraction of the LH2 complexes responded reversibly to the MIR light. MIR-induced processes might counteract MIR-independent processes to establish a closed cycle of a structural change such as the abovementioned hydrogen bond network reconstructions. This MIR response may be amplified by devising the optical process. Because the MIR response occurs before thermalization, the effect may decay within a few tens of picoseconds.^21−23^ When the picosecond pulses of the NIR and MIR light are used in the MIR experiment, the MIR response might be time-gated and amplified.^47−49^ In addition, phase-sensitive detection^50^ and a plasmonic cavity amplification using gold nanoparticles^51^ might amplify the MIR response signal.

## 5 Conclusions

For the detection of the MIR response of a single molecule from the local environment inside a protein, an intrinsic pigment at the binding site of the LH2 complex was used as a fluorescence probe. The single-molecule spectrum was different from the FTIR spectrum of the ensemble. Because the vibrational energy flow inside the protein strongly depends on the distance between the photon absorber and the probe molecule, the MIR response from the vicinity of the binding site of the single BChl-*a* pigment may be observed. Further research is essential to establish this method. Candidates for the further research systems include fluorescent cofactors in coenzymes, such as NADH and flavin.

## Supporting Information Available

Absorption spectrum of the 50 nM LH2 complex in a stock solution at 297 K and the kinetics model for the MIR-induced spectral jump of a single BChl-*a* molecule at the B800 site of the LH2 complex (PDF)

## Abbreviations

MIR: mid-infrared
NIR: near-infrared
BSA: bovine serum albumin
FTIR: Fourier-transform infrared
BChl-*a*: bacteriochlorophyll *a*
LH2 complex: light-harvesting 2 complex
fwhm: full width at a half maximum
