# Supplementary material for: Cryogenic Single-Molecule Fluorescence Detection of the Mid-Infrared Response of an Intrinsic Pigment in a Light-Harvesting Complex
Source: J Phys Chem B. 2023 May 24;127(22):4959–65. doi: 10.1021/acs.jpcb.3c00284 (PMC10258799; doi:10.1021/acs.jpcb.3c00284)
Supplement: Supplementary file 1 — jp3c00284_si_001.pdf [file jp3c00284_si_001.pdf]

Supporting information

For

"Cryogenic Single-Molecule Fluorescence Detection of the Mid-Infrared Response of an Intrinsic Pigment in a Light-Harvesting Complex"

K. Otomo, T. Dewa, M. Matsushita,* and S. Fujiyoshi*

* matsushita@phys.titech.ac.jp, fujiyoshi@phys.titech.ac.jp

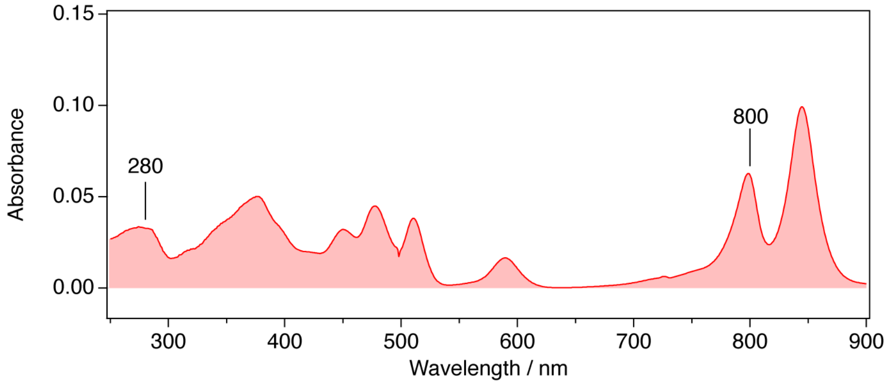

Figure S1. Absorption spectrum of the 50-nM LH2 complex in a D_2O buffer solution at 297 K.

SUPPORTING DISCUSSION

Kinetics model for the mid-infrared MIR-induced spectral jump of a single BChl *a* molecule at the B800 site of the LH2 complex.

In the present study, the mid-infrared (MIR) response spectrum of a single LH2 complex was measured using the fluorescence of a single BChl *a* molecule at the B800 site of LH2 as a probe. The response to the

MIR light was detected through the temporal behaviour of the fluorescence-excitation spectrum of the probe molecule. If we could directly observe the absorption of MIR photons by a single LH2 complex, we would obtain a spectrum of the absorption cross-section of LH2, σ(LH2). Because nuclear vibrational motion can be treated as an ensemble of independent harmonic oscillators along normal coordinates, the absorption cross-section of the whole LH2 complex is a summation of the cross-section of all of the individual vibrational modes. The vibrational modes are labelled by number, and the absorption cross-section of the i-th vibrational mode is denoted by σ^i,

$$\sigma(\mathrm{LH2}) = \sum_i \sigma^i \tag{S1}$$

In practice, the number of the vibrational modes to be summed depends on the wavenumber region of interest. In the MIR region of 1600–1700 cm^{-1}, the LH2 complex has one thousand C=O stretching vibrations as well as other types of vibrations.

In order to extract the MIR response from the NIR excitation spectrum (Fig. 3a), we chose spectral jumps between a wavenumber region above 12,620 cm^{-1} (state A) and another region below 12,565 cm^{-1} (state B). The MIR effect was quantified by the ratio of the residence time in state B (t_B) to that in state A (t_A), i.e., the residence-time ratio, $t_\mathrm{B}/t_\mathrm{A}$. From measurements of the fluorescence-excitation spectrum, the slope of $t_\mathrm{B}/t_\mathrm{A}$ against the MIR intensity (I_MIR) was determined (Figure 4) and plotted as the MIR response spectrum of a single BChl *a* molecule (Figure 5). The response spectrum is different from the FTIR spectrum of LH2, which represents σ(LH2) of eq. (S1). In order to relate this slope to the absorption cross-section, a kinetics model describing the MIR-induced spectral jumps is necessary. On this note, a simple model will be introduced, and the relation between the response spectrum and the absorption spectrum will be discussed.

The $t_\mathrm{B}/t_\mathrm{A}$ is equivalent to the ratio of the probability of finding the molecule in state B (P_B) to that in state A (P_A), i.e., $P_\mathrm{B}/P_\mathrm{A}$. The time evolution of P_B can be described by the following rate equation.

$$\frac{dP_\mathrm{B}}{dt} = k_\mathrm{A}P_\mathrm{A} - k_\mathrm{B}P_\mathrm{B} + \Phi_\mathrm{MIR}\sum_i \sigma_\mathrm{A}^i \eta_\mathrm{A}^i P_\mathrm{A} - \Phi_\mathrm{MIR}\sum_j \sigma_\mathrm{B}^j \eta_\mathrm{B}^j P_\mathrm{B} \tag{S2}$$

The equation consists of four terms; the first two terms represent MIR-independent jumps, and the third and the fourth represent MIR-dependent jumps. The MIR-independent jumps are characterized by the rate constants k_A and k_B, where k_A is a probability of making a jump in unit time from state A to B and k_B is the probability of a jump from B to A. The MIR-dependent jump occurs as one of the results of the absorption of a MIR photon by LH2. Strictly speaking, the absorption cross-section may differ from state A to state B. In eq. (S2) the cross-section of the i-th vibrational mode of state A is represented by σ_A^i and that of the j-th mode of B is represented by σ_B^j. Because the photon absorption takes place in the individual vibrational modes, the quantum efficiency with which a spectral jump occurs when a vibrational mode is excited with a MIR photon is defined for each individual vibrational mode, and designated as η_A^i for the i-th mode of state A and η_B^j for the j-th mode of state B.

The steady-state solution of the residence-time ratio is

$$\frac{t_B}{t_A} = \frac{P_B}{P_A} = \frac{k_A + \Phi_{MIR} \sum_i \sigma_A^i \eta_A^i}{k_B + \Phi_{MIR} \sum_j \sigma_B^j \eta_B^j} . \tag{S3}$$

As the spectral jumps between states A and B represent isomerization between two conformational isomers of LH2, the steady-state residence-time ratio can be regarded as an equilibrium constant of the isomerization reaction between states A and B, $K_{B \rightleftharpoons A} = [B]_{eq}/[A]_{eq}$. Because this is the exact solution to eq. (S2), the equation is valid at any intensity of MIR irradiation, from the weak-irradiation limit where the MIR effect can be expanded as a power series of the MIR intensity,

$$\frac{t_B}{t_A} = \frac{k_A}{k_B}\left\{1 + \Phi_{MIR}\left(\frac{\sum_i \sigma_A^i \eta_A^i}{k_A} - \frac{\sum_j \sigma_B^j \eta_B^j}{k_B}\right) - \Phi_{MIR}^{\ 2} \frac{\sum_j \sigma_B^j \eta_B^j}{k_B}\left(\frac{\sum_i \sigma_A^i \eta_A^i}{k_A} - \frac{\sum_j \sigma_B^j \eta_B^j}{k_B}\right) + \cdots\right\} . \tag{S4a}$$

to the strong MIR-irradiation limit where the effect saturates at

$$\frac{t_B}{t_A} = \frac{\sum_i \sigma_A^i \eta_A^i}{\sum_j \sigma_B^j \eta_B^j} . \tag{S4b}$$

The MIR intensity used in our experiments turned out to be within the linear range. A plot of t_B / t_A against I_{MIR} showed a linear relationship (Figure 4d). This means that the MIR-independent rate of k_B dominates the numerator in eq. (S3), $k_B >> \Phi_{MIR} \sum_j \sigma_B^j \eta_B^j$, which leads to

$$\frac{t_B}{t_A} = \frac{k_A}{k_B} + \frac{k_A}{k_B}\left(\frac{\sum_i \sigma_A^i \eta_A^i}{k_A} - \frac{\sum_j \sigma_B^j \eta_B^j}{k_B}\right)\frac{I_{MIR}}{h\nu_{MIR}} \tag{S5}$$

Note that the MIR irradiation is now expressed by photon energy intensity $I_{MIR} = h\nu_{MIR}\,\Phi_{MIR}$. The result of the fitting to eq. (S4) is $t_B/t_A = 0.21 + 0.0047 \times (I_{MIR}/\mathrm{W\,cm^{-2}})$. Two parameters, the intercept and the slope, are determined. The intercept $k_A/k_B = 0.21$, corresponds to the residence-time ratio measured without MIR irradiation. The slope, $\frac{k_A}{k_B}\left(\frac{\sum_i \sigma_A^i \eta_A^i}{k_A} - \frac{\sum_j \sigma_B^j \eta_B^j}{k_B}\right)\frac{1}{h\nu_{MIR}} = 0.0047\ \mathrm{W^{-1}\,cm^2}$, is variation of t_B/t_A per a unit I_{MIR} increment by 1 $\mathrm{W\,cm^{-2}}$.

Equation (S5) indicates experimentally that the ν_{MIR} dependence can be extracted only for a quantity $\sum_i \sigma_A^i \eta_A^i - \frac{k_A}{k_B}\sum_j \sigma_B^j \eta_B^j$. When the difference of the normal modes between states A and B is ignored and the value of the intercept is substituted for k_A/k_B, the expression for the MIR-response becomes a little simpler: $\sum_i \sigma^i(\eta_A^i - 0.21\eta_B^i)$. The FTIR spectrum measures the absorption cross-section of the whole LH2 $\sigma(\mathrm{LH2}) = \sum_i \sigma^i$. The MIR-response spectrum is different from the FTIR spectrum in that, for each vibrational mode, the MIR absorption cross-section is weighted by the net effect of the MIR-induced spectral jump to change the equilibrium between states A and B.

The MIR response $\sum_i \sigma^i(\eta_A^i - 0.21\eta_B^i)$ at the MIR frequency ν_{MIR} is determined from the measurement of t_B/t_A under MIR irradiation at frequency ν_{MIR} and intensity I_{MIR} by

$$\sum_i \sigma^i(\eta_A^i - 0.21\eta_B^i) \propto \left(\frac{t_B}{t_A} - 0.21\right)\frac{\nu_{MIR}}{I_{MIR}} \tag{S6}$$

The MIR-response spectrum consisting of nine different MIR frequencies is shown in Figure 5.
